# Synthetic Strategies and Biological Diversity of Biflavonoids: Current Status and Perspective

**DOI:** 10.3390/molecules31060925

**Published:** 2026-03-10

**Authors:** Yu Zhang, Yue Chai, Jiabin Wu, Shuyu Wang, Jinxin Shi, Minzhen Wei, Runhui Liu, Chunlin Zhuang

**Affiliations:** 1 The Center for Basic Research and Innovation of Medicine and Pharmacy (MOE), School of Pharmacy, Naval Medical University (Second Military Medical University), Shanghai 200433, China; zy2000815@163.com (Y.Z.); chaixiaoyue@163.com (Y.C.); 18502179238@163.com (J.W.); 19529571475@163.com (S.W.); shijinxin2001@163.com (J.S.); 2 School of Pharmacy, Ningxia Medical University, 1160 Shengli Street, Yinchuan 750004, China; 13111187554@163.com

**Keywords:** biflavonoids, synthetic strategies, chemical synthesis, biosynthesis, green synthesis, anti-inflammation, anticancer, antimicrobial activity

## Abstract

Biflavonoids are natural or pseudo-natural polyphenolic compounds formed by linking two flavonoid monomers via different bonds. Their unique dimeric structure endows them with broad-spectrum biological activities, establishing them as a core focus in drug development. However, the extremely low abundance and high structural similarity of natural biflavonoids present significant challenges. Consequently, synthetic technology has become a solution to overcome bottlenecks in supplements. The approaches involving chemical synthesis, emerging synthetic strategies, and biosynthesis are employed for the synthesis of different biflavonoids. In this review, we systematically summarize the application of diverse synthetic methods and clarify the extensive biological activities of the biflavonoids. Furthermore, we discuss the current challenges in biflavonoid synthesis and biological applications, as well as providing an outlook on future directions.

## 1. Introduction

**Figure 1:**
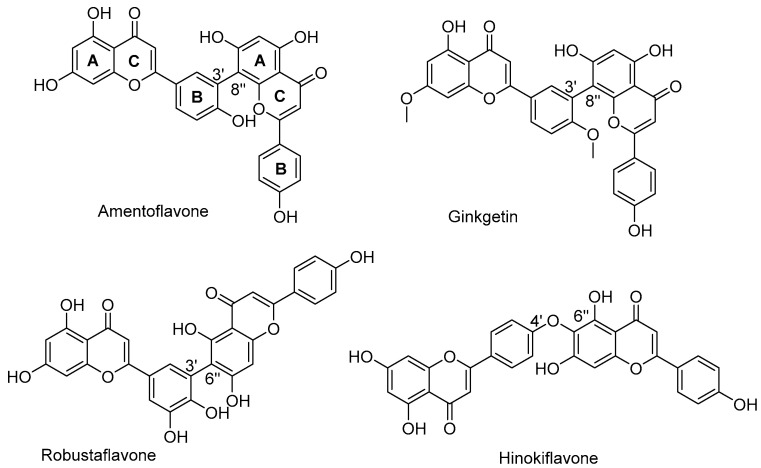
The chemical structures of representative natural biflavonoids.

Biflavonoids were first isolated from *Ginkgo biloba* by Baker et al. in 1940 [1]. By the end of 2025, more than 600 structurally diverse biflavonoids have been identified from natural sources, which are widely distributed in gymnosperms, angiosperms, bryophytes, and pteridophytes [2]. Representative natural biflavonoids include amentoflavone and ginkgetin with 3′,8″-C-C linkage, robustaflavone with 3′,6″-C-C linkage, hinokiflavone with 4′-O-6‴-C-O-C linkage, and so on. These natural biflavonoids, as a unique class of flavonoid derivatives, are dimeric polyphenolic compounds formed by two flavonoid monomers (with the 2-phenylchromone scaffold containing A, B, and C aromatic rings) linked by C-C or C-O-C bonds, and their structural diversity is mainly determined by the linkage sites of the two monomers and the types and positions of substituents such as hydroxyl and methoxy groups [3] (Figure 1).

However, biflavonoid research has long been plagued by several challenges, including extremely low natural extraction rates, harsh chemical synthesis conditions, poor water solubility (logP, 3–5), low bioavailability (oral absorption < 10%), and insufficient target selectivity [4,5]. Breakthroughs in synthetic technology are the rate-limiting step for addressing the material supply and improving bioavailability. Traditional methods for synthesizing biflavonoids are constrained by poor selectivity and low yields. Recent advances in transition metal catalysis, biocatalysis, and green chemistry have fostered a diversified paradigm. The cutting-edge progress (2024–2025) has further advanced biflavonoid research: gymnosperm-specific CYP90J enzymes were identified to drive their biosynthesis and enable microbial production of amentoflavone [6], enzymatic glycosylation of natural biflavonoids yielded derivatives with improved solubility and enhanced anti-tumor activity [7], and amentoflavone was recently found to exert anti-RSV effects via the RIG-I-MAVS pathway, expanding its clinical application potential [8]. Therefore, plenty of pseudo-natural biflavonoids with different linkage bonds are developed, further enriching the structural types of biflavonoids and greatly advancing the research on their activity. To date, biflavonoids have demonstrated multiple pharmacological activities in the treatment of neurodegenerative diseases, tumors, cardiovascular diseases, metabolic diseases, and others. This review systematically summarizes the synthetic strategies and biological activities, thereby providing a reference for the future production and clinical translation of biflavonoids. This review refers to the mainstream research on the synthesis and biological activity of biflavonoids, which were first isolated in 1990 until 2025. The inclusion criteria are peer-reviewed original research and reviews, with a focus on screening core data on synthesis methodology as well as in vivo and in vitro biological activity, excluding theoretical research without experimental verification.

## 2. Synthetic Methods of Biflavonoids

### 2.1. Chemical Synthesis

#### 2.1.1. Direct Coupling Reactions

The construction of C-C or C-O-C bonds is the key step for synthesizing biflavoniods. Direct coupling is the most straightforward method for constructing C-C or C-O-C bonds, focusing on selective activation of specific positions of flavonoid monomers. The current mainstream methods include Ullmann condensation and Suzuki cross-coupling reactions.

**(1) Ullmann Condensation**

**Figure 2:**
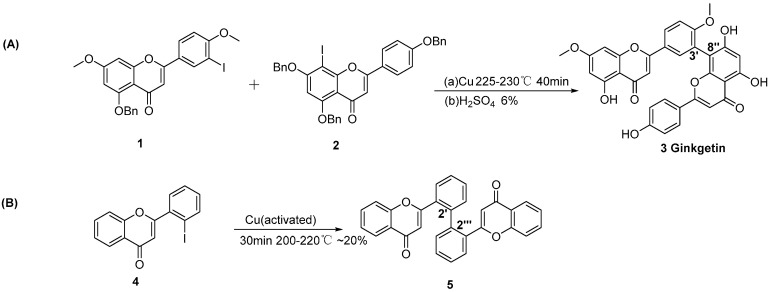
Synthesis of ginkgetin (**A**) and 2′,2‴-linkage biflavonoids (**B**) by Ullmann condensation.

In 1965, ginkgetin was first synthesized by Nakazawa et al. using the Ullmann condensation of 3′-iodoflavone with 8″-iodoflavones, subsequently followed by deprotection (Figure 2A) [9]. This method yielded ginkgetin (3′,8″-linkage) in a 6% yield. The coupling reaction relies on copper-catalyzed coupling of iodoflavones at high temperatures (200–230 °C). Although this reaction does not require complex ligands, the reaction suffers from poor site selectivity, often generating homocoupling by-products (e.g., 3′-3′ and 8-8″) as well as oxidative polymerization products of flavonoids induced by high temperatures. Therefore, the Ullmann reaction is proposed to be suitable for the synthesis of symmetrical biflavonoids. For instance, Sagrera et al. successfully synthesized 2′,2‴-linkage biflavonoids using Ullmann condensation (Figure 2B) [10].

**Figure 3:**
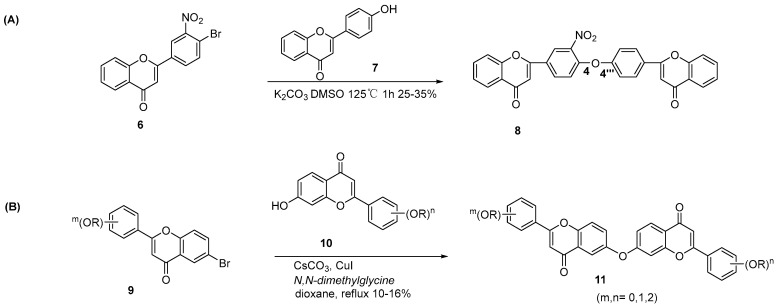
Synthesis of 4′-O-4‴-linkage (**A**) and 6-O-7″-linkage C-O-C biflavonoid derivatives (**B**) by Ullmann condensation.

The Ullmann reaction is also a standard strategy for synthesizing C-O-C-bonded biflavonoids via nucleophilic substitution between a phenolic hydroxyl and an aryl halide. In 1970, Chen et al. reported the direct coupling of 4′-hydroxyflavones with 4′-haloflavones via Ullmann condensation (Figure 3A) [11]. In 2009, Che et al. synthesized 6-O-7″-linkage C-O-C biflavonoids via intermolecular Ullmann etherification (Figure 3B) [12]. The coupling of 6-bromoflavone and 7-hydroxyflavone in anhydrous dioxane, using CuI (20 mol%), N,N-dimethylglycine (8 mol%), and Cs_2_CO_3_ (8 mol%) at 100 °C for 24 h, afforded 8 methoxy-substituted biflavonoids in 10–16% yield. Subsequent demethylation with BBr_3_ in chloroform at 0 °C and recrystallization provided 8 hydroxy-substituted derivatives in 65–75% yield. Applying the reaction, 17 biflavonoid derivatives were generated.

**(2) Suzuki cross-coupling reactions**

For the asymmetric biflavonoids, Suzuki cross-coupling has emerged as the mainstream approach for the site-specific synthesis of C-C bond-linked biflavonoids [13]. This reaction involves the palladium-catalyzed coupling of flavonoid pinacol boronates or boronic acids with haloflavones (iodo or bromo derivatives), mainly using Pd catalysts and phosphine ligands (e.g., PPh_3_, XPhos) [14]. This reaction proceeds at moderate temperatures in common solvents with high regioselectivity and yields of 30–60%, thus overcoming the poor site selectivity inherent to the Ullmann reaction.

**Figure 4:**
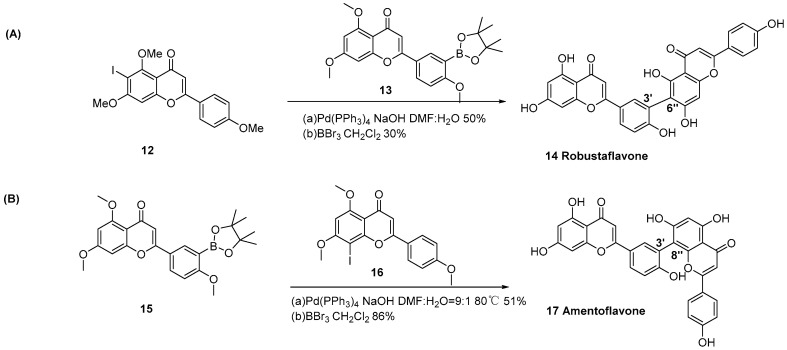
Synthesis of Robustaflavone (3′,6″-linkage) (**A**) and Amentoflavone (3′,8″-linkage) (**B**) by Suzuki cross-coupling reactions.

Zembower et al. achieved the first total synthesis of Robustaflavone (3′,6″-linkage) [15]. Key intermediates were prepared via thallium-assisted C-6 iodination and palladium-catalyzed borylation, followed by Suzuki coupling and BBr_3_-mediated deprotection to yield the target product (Figure 4A). Park et al. accomplished the first synthesis of Amentoflavone (3′,8″-linkage) using 3′- flavonoid pinacol boronate and 8-iodoflavone as the key intermediates via Suzuki coupling followed by deprotection with BBr_3_ (Figure 4B) [16].

**Figure 5:**
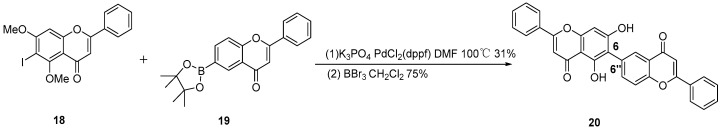
Synthesized 6,6″-linked biflavonoid by Suzuki cross-coupling reaction.

**Figure 6:**
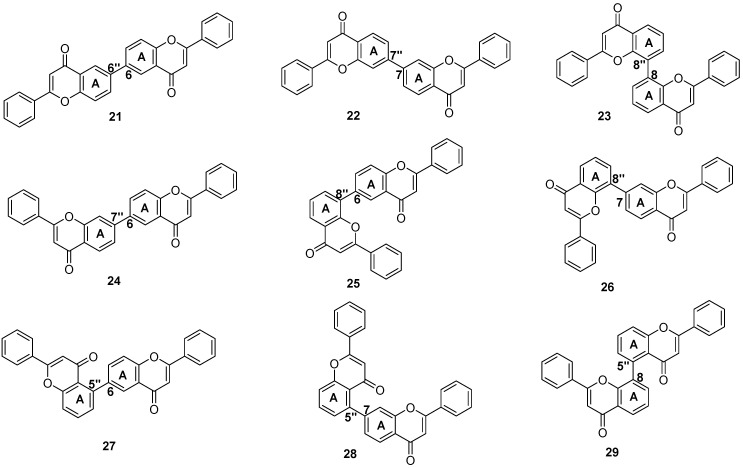
Synthesis of the A ring–A ring-linked biflavonoids by Suzuki cross-coupling reactions.

Biflavonoids bearing A ring–A ring linkage are an exceedingly rare class of compounds in natural sources, which is mainly attributed to the regioselectivity of biosynthetic enzymes rather than the simple steric hindrance or electron density effects of the flavonoid skeleton. The natural flavonoid dimerases exhibit strict substrate specificity and catalytic regioselectivity, which rarely catalyze the C-C coupling between the A rings of two flavonoid monomers. In synthetic chemistry, Suzuki coupling is a feasible strategy for constructing the A ring-A ring linkage biflavonoids. Lim et al. synthesized the 6,6″-linkage biflavonoid by subjecting 6-iodo-5,7-dimethoxyflavone and a 6-flavonoid pinacol boronate to Suzuki coupling, with subsequent deprotection (Figure 5) [17]. Kohari et al. developed an efficient synthetic method based on Suzuki cross-coupling, which is specifically designed for the preparation of A ring–A ring-linked biflavonoids (both symmetrical and asymmetrical types) (Figure 6) [18]. Starting from 6-, 7-, and 8-bromoflavones, the corresponding 6-, 7-, and 8-flavonoid pinacol boronates were obtained via palladium-catalyzed pinacol boronation with bis(pinacolato)diboron (pin_2_B_2_). Subsequent coupling of 8-flavonoid pinacol boronates with bromoflavones or flavon-5-yl trifluoromethanesulfonate yielded a series of biflavonoids (symmetrical **21**–**23**, asymmetrical **24**–**26**). Furthermore, the cross-coupling of flavonoid pinacol boronates with another intermediate flavon-5-yl trifluoromethanesulfonate efficiently afforded a range of asymmetrical biflavonoids, including 5″,6 -linkage **27**, 5″,7-linkage **28**, and 5″,8-linkage **29**, with high yields (90–93%).

**Figure 7:**
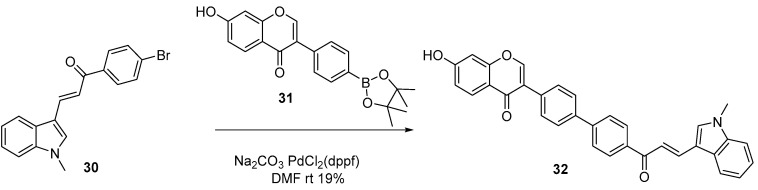
Synthesis of the “hybrid” biflavonoids by Suzuki cross-coupling reactions.

The other advantages of the Suzuki reaction is its ability to expand the scope of reactions, enabling the synthesis of uncommon biflavonoid architectures. First, Suzuki coupling is not limited to the 2-phenylchromone scaffold. Sum et al. successfully synthesized a class of rare “hybrid” biflavonoids using flavonoid-derived pinacol boronates and bromoflavonoids as intermediates (Figure 7) [19]. These hybrids are constructed from monomers of distinct flavonoid subclasses, such as isoflavone-flavones and isoflavone-chalcones.

**Figure 8:**
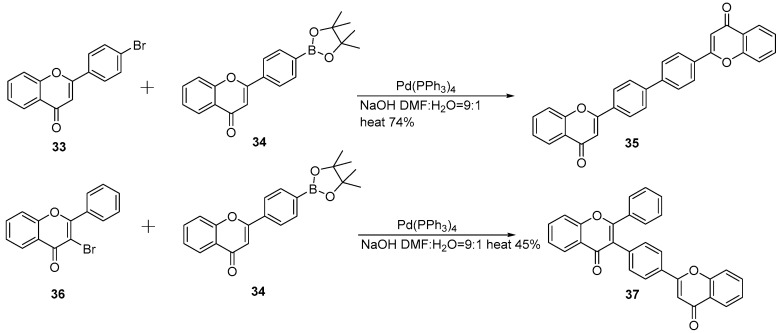
Synthesis of 4′,4‴-linkage and 3,4‴-linkage biflavonoids by Suzuki cross-coupling reactions.

Second, the electronic properties and steric hindrance of substituents around the reaction site significantly influence the feasibility and yield of the reaction. Chen et al. synthesized flavonoid pinacol boronate by reacting 4-bromoflavone with bis(pinacolato)diboron under the catalysis of PdCl_2_(dppf) and K_2_CO_3_ in DMF at 90 °C (Figure 8). The resultant flavonoid pinacol boronate was then subjected to coupling with 4′- and 3-bromoflavones in DMF/water (9:1) at 90 °C using Pd(PPh_3_)_4_ and NaOH as the catalytic system, affording biflavonoid **35** (4′,4‴-linkage) in 74% yield and biflavonoid **37** (3,4‴-linkage) in 45% yield, respectively [20,21].

#### 2.1.2. Precursor Cyclization Reaction

Unlike the aforementioned direct coupling, the precursor cyclization strategy typically starts from one or more pre-modified precursor molecules and constructs the core scaffold of biflavonoids through intramolecular or intermolecular cyclization reactions in one or several steps, including Sonogashira coupling reactions, Stille coupling reactions, and other strategies. The methods exhibit unique advantages in constructing complex biflavonoids with specific linkage patterns [22].

**(1** **) Sonogashira coupling reactions**

Sonogashira coupling is to conjugate one flavonoid unit to an aromatic compound at a specific site via an alkynyl group, generating key alkynyl-containing intermediates. Subsequent cyclization and oxidation then construct the C-C bond framework of the target biflavonoids. This strategy is particularly suitable for the synthesis of biflavonoids that are typically difficult to achieve via conventional coupling reactions. It also effectively addresses the challenge of site-specific coupling at sterically hindered positions and heterocyclic fragment ligation.

**Figure 9:**
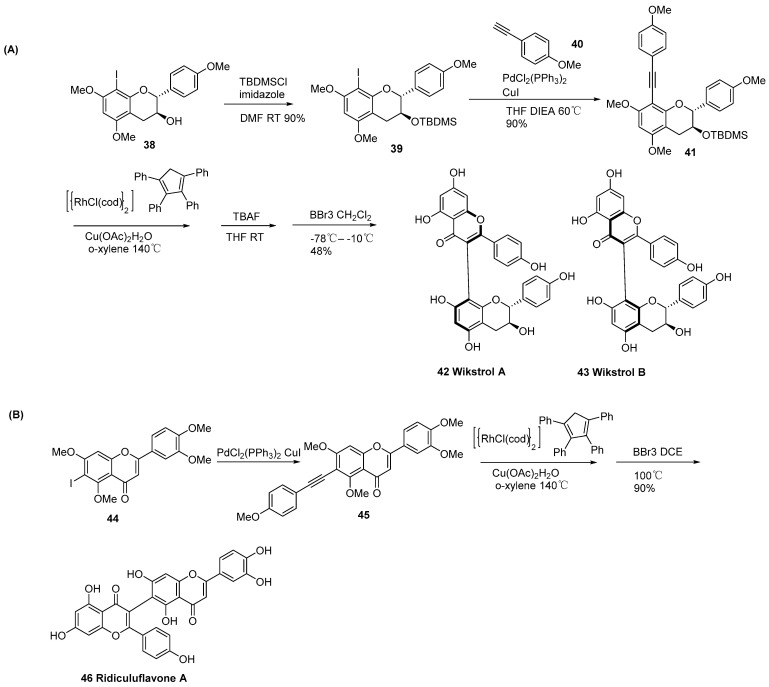
Synthesis of Wikstrol A, B (**A**) and Ridiculuflavone A (**B**) by Sonogashira coupling reactions.

Lu et al. adopted a strategy centered on “stepwise construction of intermediates followed by key coupling” (Figure 9A) [23]. Compound **38**, whose hydroxyl group is protected as a silyl ether prior to Sonogashira coupling with 4-ethynylanisole to yield compound **41** Rhodium-catalyzed oxidative cyclization of **41** with 2-hydroxy-4,6-diisopropoxybenzaldehyde, followed by desilylation, gives key intermediates. Finally, demethylation with BBr_3_ affords the target products Wikstrol A (**42**) and Wikstrol B (**43**) in 50% and 48% yields, respectively [24]. Furthermore, this author also successfully synthesized Ridiculuflavone A, which shares a structural similarity with Wikstrol (Figure 9B) [23].

**(2) Stille coupling reactions**

**Figure 10:**
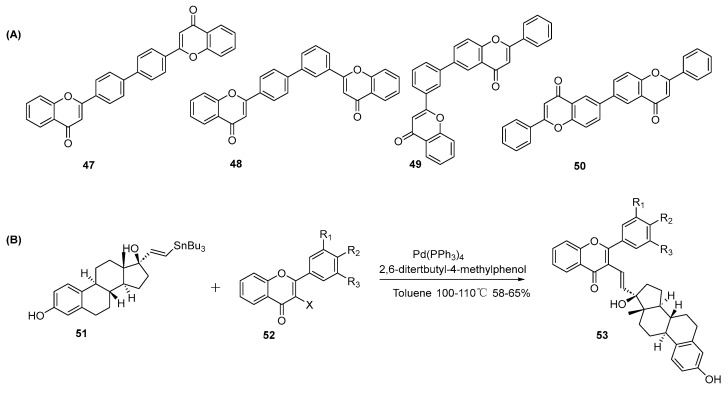
Synthesis of 47–50 (**A**) and 53 (**B**) by Stille coupling reactions.

Stille coupling is also applied in biflavonoid synthesis using a stannum intermediate. Chen and co-workers first prepared tributylstannylflavones by reacting 4′-, 6-, and 3′-bromoflavones with hexabutylditin, using Pd(PPh_3_)_4_ as the catalyst under reflux conditions in toluene [21]. The resultant tributylstannylflavones were then subjected to coupling with bromoflavones substituted at the 3-, 6-, 3′-, and 4′-positions, yielding biflavonoids **47**–**50** with yields ranging from 25% to 50% (Figure 10A). A similar application was reported to have achieved the site-directed conjugation of flavones with estradiol, resulting in the synthesis of flavone–estradiol conjugates (Figure 10B) [25].

**(3) Other strategies**

**Figure 11:**
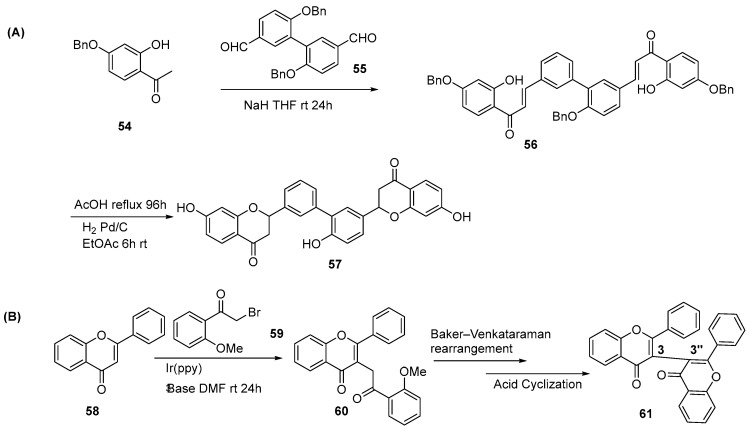
Two examples, (**A**) and (**B**), for the synthesis of biflavonoids by other reactions.

When Ullmann condensation of two iodoflavone units is unfeasible or predominated by side reactions, a “precursor-dimerization” strategy provides a viable alternative. Sagrera and co-workers accessed biphenyl derivatives via Ullmann coupling, followed by aldol condensation, acid-mediated cyclization, and deprotection to afford a series of symmetrical biflavonoids (Figure 11A) [10].

López et al. reported the first synthesis of an unsymmetrical 3-3″ -biflavonoid using Ir(ppy)_3_ to catalyze the coupling of a flavone monomer with an alkyl bromide [26]. The resulting product served as the precursor for a subsequent Baker–Venkataraman rearrangement, which, upon cyclization under refluxing sulfuric acid/methanol, afforded the target biflavonoid (Figure 11B).

As a classic method for C-C bond formation, Ullmann coupling features a simple reaction system without using complex catalyst ligands. However, its harsh conditions, typically high temperature, along with low yields and poor regioselectivity, limit its application in biflavonoid synthesis. In contrast, Suzuki coupling has become the most widely used method for preparing symmetrical and asymmetrical biflavonoids at various substitution sites. It offers high functional group tolerance, including compatibility with methoxy- and hydroxyl-protecting groups, and uses relatively non-toxic boronic acids or esters. The reactions generally provide stable yields that meet the structural requirements of most biflavonoid frameworks. Sonogashira coupling (Pd/Cu dual catalysis) enables site-directed coupling via alkynyl groups, making it suitable for preparing alkynyl-containing biflavonoid precursors. Nevertheless, this strategy requires an additional transformation step and relies on alkynyl reagents. Although Stille coupling exhibits good functional group tolerance, the high toxicity of stannane reagents, significant steric hindrance effects on transmetalation, and common side reactions (e.g., reduction in haloflavones) severely restrict its practical use. Overall, these coupling significantly outperforms its advantages and disadvantages in terms of generality, safety, yield, and operational simplicity, and it is capable of meeting the synthetic demands of the vast majority of biflavonoids.

### 2.2. Emerging Synthetic Methods

#### 2.2.1. Oxidative Coupling

**Figure 12:**
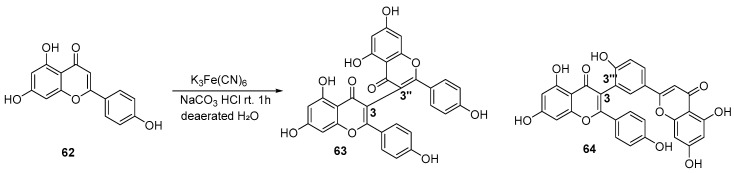
Synthesis of the 3,3″-linkage and 3,3‴-linkage biflavonoids by K_3_Fe(CN)_6_ oxidation.

Oxidative coupling is capable of efficiently constructing the key C-C or C-O-C linkages between flavonoid monomers by mimicking the core mechanism of phenolic hydroxyl oxidation-radical coupling involved in the biosynthesis of bioflavonoids [27]. Currently, research on the oxidative synthesis of biflavonoids mainly focuses on the optimization of oxidant systems and the construction of new catalytic systems. Oxidants include chemical oxidants (e.g., ceric ammonium nitrate, ferric chloride) and green oxidants (e.g., oxygen, air); catalytic systems encompass metal ion catalysis, enzyme catalysis, electrocatalysis, and photocatalysis. Molyneux and co-workers achieved the dimerization of apigenin under the oxidative catalytic conditions of sodium carbonate and potassium ferricyanide K_3_Fe(CN)_6_ (Figure 12) [28].

**Figure 13:**
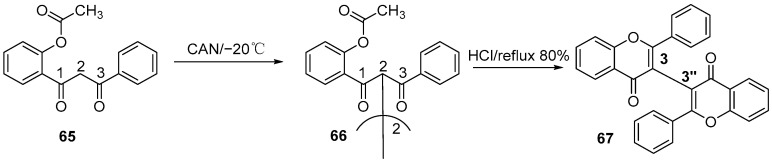
Synthesis of the 3,3″-linkage biflavonoids by CAN oxidation.

In 2015, Nanjan et al. developed an efficient one-pot synthesis strategy using ceric ammonium nitrate (CAN) as the single-electron transfer oxidant (Figure 13) [29]. This strategy first mediated the oxidative dimerization of polysubstituted diaryl 1,3-diketones to afford tetraketone intermediates, and the double cyclodehydration was accomplished with 10% methanolic hydrochloric acid under reflux.

**Figure 14:**
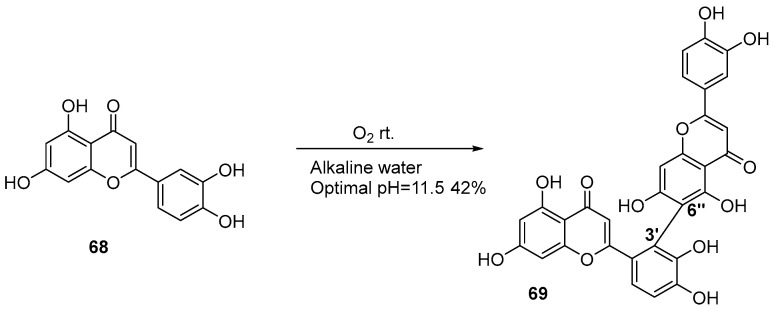
Synthesis of the 3′,6″-linkage biflavonoids by oxygen-mediated oxidation.

Green oxidants, such as oxygen and air, instead of traditional chemical oxidation, are applied in oxidative coupling. In 2022, Huang group developed an oxygen-mediated oxidative coupling strategy (Figure 14) [30]. In this protocol, molecular oxygen served as both the oxidant and hydrogen atom acceptor, enabling the oxidative coupling of flavonoids in an alkaline aqueous system. Under the optimized conditions, luteolin underwent homocoupling to achieve 10 g-scale synthesis in 42% yield at room temperature.

#### 2.2.2. Electrocatalytic Synthesis

**Figure 15:**
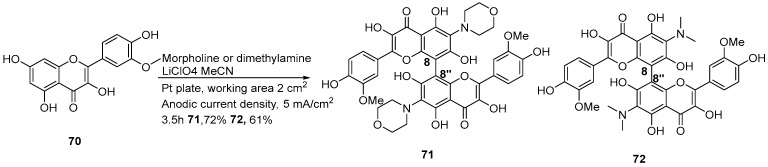
Synthesis of the 8,8″-linkage biflavonoids by electrocatalytic synthesis.

Electrocatalytic synthesis is a green strategy that avoids toxic reagents via electron transfer. Its mild conditions and controllable selectivity make it highly promising for constructing complex flavonoid derivatives. In 2014, Nadirov et al. developed an electrocatalytic synthesis method for biflavonoid amino derivatives (Figure 15) [31]. Using isorhamnetin as the starting material, morpholine and dimethylamine as amine sources, acetonitrile as the solvent, and lithium perchlorate as the electrolyte, electrolysis was performed for 3.5 h in a divided cell with a platinum anode. This protocol successfully afforded two types of 6,6′-diamino-substituted 8-8″-linked biflavonoid amino derivatives. The amino groups were successfully introduced while the 8-8″ linkages were simultaneously formed.

### 2.3. Biosynthetic Strategies

Biosynthesis, by deciphering the natural synthetic pathways of biflavonoids and leveraging enzyme-catalyzed reactions for green and efficient production, has emerged as a key direction to overcoming the bottlenecks of chemical synthesis in recent years. Its core advancements focus on the identification of key polymerases, elucidation of synthetic pathways, and establishment of microbial production platforms.

**Figure 16:**
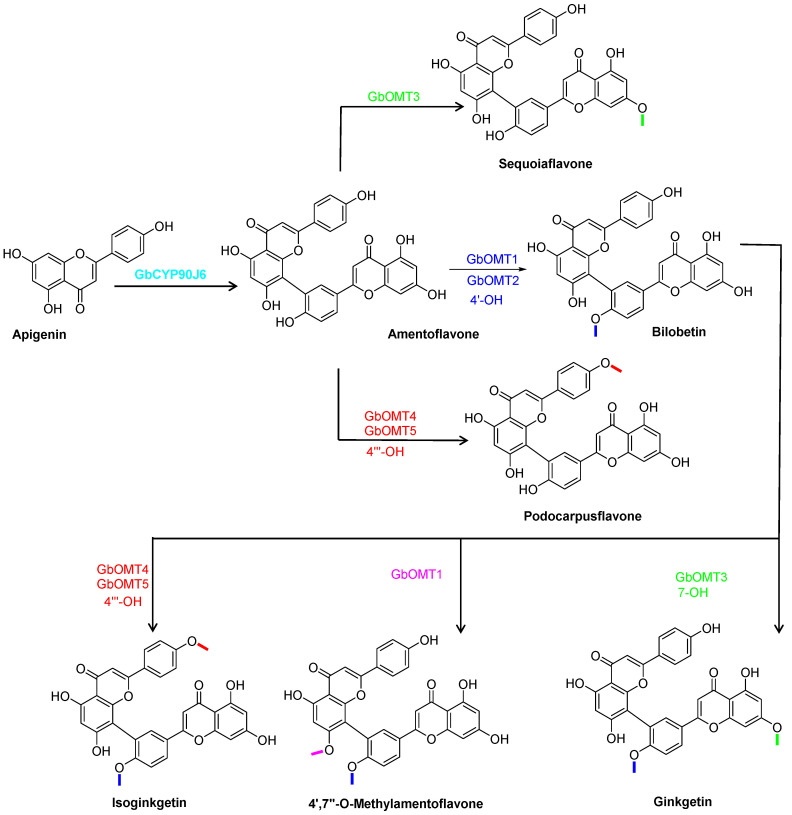
The synthetic pathways of 3′,8″- linked biflavonoids.

In 2025, Wang group first identified gymnosperm-derived CYP90J subfamily enzymes that catalyze C-C bond formation in bioflavonoids [6]. They further elucidated the complete biflavonoid biosynthetic pathway (from dimerization to methylation modification), its gymnosperm-specific evolutionary origin, and the molecular mechanism regulating precise regioselectivity. They identified GbCYP90J6, a gymnosperm-specific cytochrome P450 enzyme, to efficiently catalyze apigenin dimerization to amentoflavone. With the key intermediate amentoflavone, five O-methyltransferases (GbOMT1-GbOMT5) perform regioselective modification, generating a variety of natural biflavonoids including sequolaflavone, bilobetin, podocarpusflavone, ginkgetin, isoginkgetin, and 4′,7″-O-methyl amentoflavone, respectively (Figure 16). Among these enzymes, GbOMT1 preferentially targets the 4′-OH and 7″-OH sites, GbOMT2 specifically modifies the 4′-OH site, GbOMT3 targets the 7-OH site, and GbOMT4 and GbOMT5 selectively modify the 4‴-OH site.

**Figure 17:**
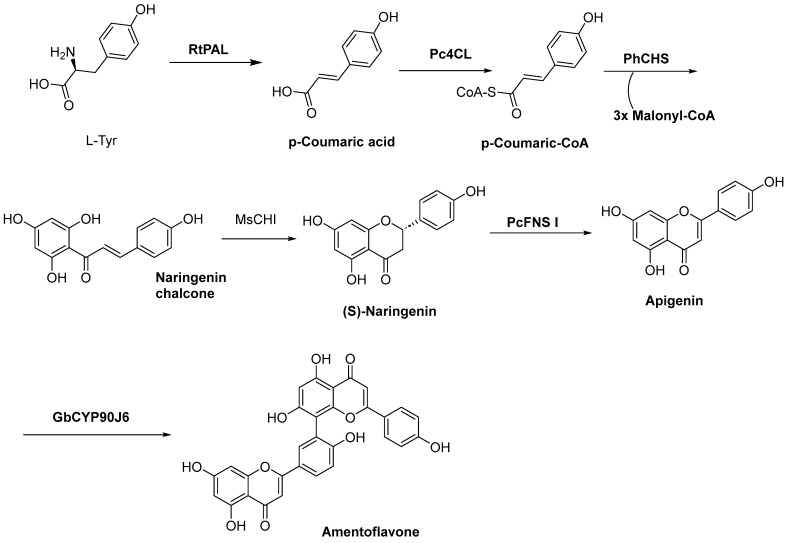
Biosynthetic modules for Amentoflavone production from L-Tyr in GJA3 strain.

They further confirmed the biosynthetic modules for amentoflavone (Figure 17). The research constructed an engineered *Escherichia coli* strain, GJA3, with high-level production of L-tyrosine. By further assembling the “apigenin biosynthesis module” and the “dimerization module”, the de novo biosynthesis of amentoflavone was successfully achieved, with a shake-flask fermentation titer of 4.75 mg/L. First, L-tyrosine undergoes deamination catalyzed by phenylalanine ammonia-lyase (RfPAL) to yield p-coumaric acid. Subsequently, p-coumaric acid is activated by 4-coumarate: CoA ligase (Pc4CL) to form p-coumaroyl-CoA. Next, p-coumaroyl-CoA undergoes a condensation reaction with three molecules of malonyl-CoA under the catalysis of chalcone synthase (PhCHS), producing naringenin chalcone. Naringenin chalcone is then cyclized and isomerized to (S)-naringenin by chalcone isomerase (MsCHI), which is further oxidized and dehydrated by flavone synthase I (PcFNS I) to generate apigenin. Finally, two molecules of apigenin undergo oxidative coupling catalyzed by the flavonoid dimerization enzyme (GbCYP98J6) to afford the biflavonoid compound amentoflavone.

## 3. Biological Activity of Biflavonoids

Tremendous effort has been devoted to exploring the biological activities of biflavonoids, particularly natural bifavonoids. The activities of biflavonoids mainly include anti-tumor activity, anti-Alzheimer’s disease, anti-inflammatory activity, and antimicrobial activity (Table 1, Table 2, Table 3 and Table 4).

### 3.1. Anti-Tumor Activity

Amentoflavone exhibits broad anti-tumor activity. Jung et al. reported that amentoflavone effectively suppressed A549 lung cancer xenografts in vivo [32]. Its mechanism involves potentially acting as a potent inhibitor of the detoxification enzyme AKR1B10 (IC_50_ = 1.54 μM), blocking doxorubicin metabolism to reverse chemoresistance and synergizes with the drug in AKR1B10-high cells (CI, 0.58–0.86). Sun et al. reported its induction of ferroptosis via the ROS/AMPK/mTOR pathway in endometrial carcinoma, downregulating anti-ferroptotic proteins (SLC7A11, GPX4) and proliferation markers (Ki67, PCNA), thereby inhibiting KLE cell viability and promoting apoptosis [33]. Delicaflavone (DLF), a biflavonoid isolated from *Selaginella* species, exhibited potent anti-tumor activity against colorectal cancer (CRC) [34]. In vitro, DLF suppressed the proliferation of HT29 and HCT116 cells, induced G2/M phase arrest, and promoted ROS accumulation and apoptosis, while concomitantly inhibiting the PI3K/AKT/mTOR and Ras/MEK/ERK signaling pathways. In vivo, DLF achieved a 55.2% tumor inhibition rate in HT29 xenografts by upregulating Caspase-3/9 and downregulating CD34/Ki67, thereby exerting pro-apoptotic and anti-angiogenic effects. Isoginkgetin was reported to exert distinct anticancer activity by inhibiting pre-mRNA splicing with an IC_50_ of 30 μM [35]. Hinokiflavone (HF) was reported to inhibit esophageal squamous cell carcinoma (ESCC) cell proliferation, migration, and invasion by suppressing the PI3K/AKT/mTOR pathway and downregulating MMP2/9 [36]. In vivo models, HF significantly inhibited ESCC xenograft tumor growth with minimal toxicity to major organs at a dose of 50 mg/kg. Li group synthesized Amantoflavone 7-O-β-D-glucoside by an enzymatic synthesis strategy of UGT74AN2 coupled with AtSuSy. The water solubility of the derivative was 22.6 times higher than that of natural Amantoflavone, and its anti-proliferative activity against prostate cancer PC-3 cells was significantly enhanced (IC_50_ = 57.7 μM vs. natural 102.2 μM), without normal cardiomyocyte toxicity. The result directly confirmed that synthetic modification can simultaneously optimize the drug properties and anti-tumor activity of the bioflavonoids [7].

### 3.2. Anti-Alzheimer’s Disease

Several amentoflavone-type biflavonoids (e.g., amentoflavone, sequiaflavone and bilobetin) were reported to depolymerize Aβ_42_ fibrils [37]. The core mechanism of biflavonoid-induced Aβ_42_ fibril disaggregation is their specific interaction with Aβ_42_ fibrils: biflavonoids first bind preferentially to the aromatic-rich N-terminal pocket (residues 4–14) of Aβ_42_ fibrils via π-π stacking between their planar aromatic rings and Aβ_42_’s aromatic residues (Phe4, His6, Tyr10). Their hydroxyl groups then form hydrogen bonds with the Aβ_42_ fibril’s peptide backbone, competing with the intermolecular hydrogen bonds that stabilize the β-sheet structure of Aβ_42_ aggregates. This dual interaction reduces Aβ_42_’s β-sheet content (from 35 to 20%) and triggers the conformational collapse of fibrils into non-toxic amorphous aggregates. Amentoflavone was most potent (EC_50_ = 0.70 ± 0.16 μM), while sequiaflavone was weakest (EC_50_ = 12.5 ± 5.3 μM), indicating the importance of the hydroxyl groups. Biflavonoids also exhibited structure-dependent inhibitory activity against Aβ_40_ aggregation. Kinoshita and co-workers reviewed the structure–activity relationships (SARs) of various biflavonoid scaffolds in this regard [38]. The thioflavin-T (Th-T) fluorescence assays showed that amentoflavone and its monomethoxyl derivatives (sequoiaflavone, podocarpusflavone A and Bilobetin) inhibited Aβ_40_ aggregation (IC_50_≈5 μM), comparable to myricetin (4.1 ± 0.4 μM). In amentoflavone-type biflavonoids, retaining two or more free hydroxyl groups at the 7, 4′, 7″, and 4‴ positions is crucial for maintaining potent activity. The reason was proposed that hydroxyl groups can stabilize interactions with Aβ_40_ through hydrogen bonding. In the model of porcine brain capillary endothelial cells (BCEC) in vitro, Gutmann et al. found that amentoflavone could have the ability to cross the blood–brain barrier (BBB), with an apparent permeability coefficient P_app_ of 4.59 × 10^−6^ cm/s [39]. This evidence indicates that biflavonoids have potential brain penetrating pharmacokinetic benefits in the therapy of Alzheimer’s disease.

Structurally, not limited to biflavonoids, biflavonoid glycosides are also among the potential natural active molecules that inhibit Alzheimer’s disease-related enzymes. Kouam group isolated and purified two biflavonoid compounds, (2S,3S)-volkensiflavone-7-O-β-glucopyranoside and (2R,3S)-volkensiflavone-7-O-β-D-acetylglucopyranoside, from the stem bark of *Allanblackia floribunda* and found that they were triple inhibitors of monoamine oxidase A (MAO-A), β-secretase (BACE-1), and glycogen synthase kinase-3β (GSK-3β) [40]. (2S,3S)-volkensiflavone-7-O-β-glucopyranoside and (2R,3S)-volkensiflavone-7-O-β-D-acetylglucopyranoside were found to be reversible and moderately selective MAO-A inhibitors with IC_50_ of 35.85 ± 0.03 μM and 25.54 ± 0.05 μM, respectively. These compounds strongly inhibited BACE1 (IC_50_ = 2.48 ± 0.11 μM and 2.50 ± 0.17 μM, respectively) and GSK-3β (IC_50_ = 9.39 ± 0.06 μM and 7.17 ± 0.09 μM, respectively). Furthermore, molecular docking showed that both compounds can bind to the active sites of the enzymes, providing potential candidate drugs for the multi-target treatment of Alzheimer’s disease.

### 3.3. Anti-Inflammatory Activity

Singab et al. reported robustaflavone and amentoflavone exhibiting significant anti-inflammatory activities [41]. They suppressed the generation of superoxide anion with an IC_50_ value of 1.01 μM, and inhibited elastase release with IC_50_ values of 0.45 μM (robustaflavone) and 0.75 μM (amentoflavone), respectively. Molecular docking suggested their binding to human neutrophil elastase (HNE), supporting potential applications in inflammation-related diseases. Shim et al. identified that hinokiflavone and 7″-O-methyl hinokiflavone exerted significant anti-inflammatory activity using lipopolysaccharide (LPS)-induced RAW 264.7 macrophage and HT-29 colon epithelial cell models, with no cytotoxicity at 1–10 μM [42]. Hinokiflavone (HF) was also reported to alleviate APAP-induced liver injury in vitro and in vivo via the SIX4/Akt/Stat3 pathway [43]. Gill and co-workers identified that morelloflavone exhibits significant anti-inflammatory activity [44]. This compound irreversibly inhibited group II and III secretory phospholipase A_2_ (sPLA_2_), with IC_50_ values of 0.9 μM for human recombinant synovial sPLA_2_ and 0.6 μM for bee venom sPLA_2_, while showing no effect on cytosolic PLA_2_. Park and co-workers investigated the effects of various biflavonoid derivatives with different configurations on the production of prostaglandin E_2_ (PGE_2_) and nitric oxide (NO) in lipopolysaccharide (LPS)-stimulated RAW 264.7 macrophages [45]. In vitro, the 6,6″- linked biflavonoid derivative exhibited the most potent inhibitory activity, with an IC_50_ value of 3.7 μM (compared to 20.7 μM for ginkgetin). Its mechanism of inhibition involves the direct suppression of COX-2 activity rather than the downregulation of its expression, and it showed no significant inhibitory effect on iNOS-mediated NO production. In vivo, intraperitoneal administration of the 6,6″-linkage biflavonoid derivative at a dose of 5 mg/kg demonstrated 22.2% anti-inflammatory activity against carrageenan-induced paw edema in rats.

Biflavonoids also have extensive applications in the field of anti-neuroinflammation. Heneka group reported that 7,7′-dimethylophorbol can inhibit the secretion of NO in a concentration-dependent manner in the neuroinflammatory model induced by LPS and nigricin [46]. It significantly reduced the secretion levels of pro-inflammatory cytokines IL-1β and IL-18. It could effectively restore the damaged Aβ phagocytic function after the activation of the inflammasome. Agathisflavone, as a type of dimeric flavonoid, exhibited prominent neuroinflammatory and neuroprotective activities [47]. It inhibited the proliferation of microglia by reducing the proportion of Iba-1^+^/BrdU^+^ cells, lower the expression of M1-type microglia markers CD68 and NF-κB, blocked their activation towards a pro-inflammatory phenotype, and had no significant adverse effects on astrocytes.

### 3.4. Antimicrobial Activity

Biflavonoids exhibit a broad range of antimicrobial activities, including antibacterial, antifungal, and antiviral activities. Zhang group found that ginkgetin exerted prominent anti-biofilm activity and antibacterial synergy against *Escherichia coli* [48]. In the concentration range of 6.25~100 µM, ginkgetin reduced biofilm formation in a dose-dependent manner and suppressed bacterial motility by decreasing the production of extracellular polymeric substances (EPS). Ginkgetin inhibited the synthesis of the AI-2 signaling molecule with an IC_50_ of 22.33 µM, as well as significantly downregulating the transcription of fimbriae-associated genes (e.g., *csgA*, *csgD*), flagellar formation genes (e.g., *flhC*, *flhD*, *fliC*, *fliM*), and quorum sensing (QS)-related genes (e.g., *luxS*, *lsrB*, *lsrK*, *lsrR*). Tetrahydroamentoflavone, which was similar to the structure of ginkgetin (3′,8″-linkage), was isolated and identified from the fruits of the Brazilian peppertree (*Schinus terebinthifolius*) [49]. Tetrahydroamentoflavone exhibited the strongest activity, with a minimum inhibitory concentration (MIC) as low as 0.063 mg/mL and a minimum bactericidal concentration (MBC) ranging from 0.063 to 0.125 mg/mL against Gram-positive bacteria, such as *Bacillus subtilis* and *Staphylococcus carnosus*. Tetrahydroamentoflavone also inhibited biofilm formation, achieving a 99% inhibition rate against *Staphylococcus carnosus*. The Musman group isolated and identified a novel biflavonoid, macrophylloflavone, from *Garcinia macrophylla* Mart [50]. In vitro assays demonstrated that this compound exhibited potent antibacterial activity against the standard strains of *Escherichia coli* and *Staphylococcus aureus*.

In terms of antiviral activity, Takahashi group screened bioflavonoids for anti-influenza activity and identified that ginkgetin exhibited influenza virus sialidase inhibitory activity [51]. Ginkgetin-sialic acid conjugates were synthesized and the deacetylated derivative (8R, 8S) showed stronger anti-sialidase activity against H1N1/H3N2 than F36, with no cytotoxicity. In vivo studies revealed that these conjugates could significantly prolong the survival time of mice infected with H1N1 influenza virus, and the survival rate of mice treated with 8R reached 62.5% on day 21 post-infection. These findings supported that ginkgetin-sialic acid conjugates possess potent and low-toxic anti-influenza virus activity both in vitro and in vivo. Feng group focused on the therapeutic effect and mechanism of amentoflavone on pulmonary inflammatory injury induced by respiratory syncytial virus (RSV) [8]. In vitro and in vivo experiments demonstrated that amentoflavone significantly inhibited RSV replication and alleviated pulmonary inflammatory damage. The underlying mechanisms were proposed to include direct activation of the RIG-I-MAVS innate immune signaling pathway, promotion of type I interferon (IFN-α/β) release, and upregulation of interferon-stimulated gene (ISG) expression.

Lee and co-workers found that isocryptomerin exhibited significant antifungal activity, with a MIC of 18.11 μM against *Candida albicans*, *Geotrichum candidum*, and *Saccharomyces cerevisiae* [52]. Compared to the positive drug amphotericin B, isocryptomerin exhibited only 11.7% hemolysis at the highest concentration (36.23 μM) and showed no hemolytic activity at other tested concentrations, indicating superior safety. Flow cytometry, protoplast regeneration assays, and fluorescence anisotropy assays suggested that this compound can cause depolarization of *Candida albicans* cell membranes, interfering with membrane fluidity.

## 4. Challenges and Perspectives

Biflavonoids, with their unique chemical structures and broad-spectrum biological activities, exhibit enormous research value and application potential in the fields of natural medicinal chemistry, pharmacology, and drug development [53,54]. Currently, both the optimization of chemical synthesis processes for biflavonoids and the in-depth exploration and clinical translation of their biological activities are confronted with numerous bottlenecks that urgently need to be broken through.

### 4.1. Chemical Synthesis

The chemical synthesis of biflavonoids represents a major bottleneck for their large-scale application, with core challenges lying in selectivity control, reaction efficiency, and process feasibility. The challenge lies in overcoming the structural similarity of multiple phenolic hydroxyl groups and aromatic C-H bonds in flavonoid monomers to achieve regioselectivity and chemoselectivity [55,56,57].

The Ullmann coupling is usually appliable under high temperature and strong alkaline conditions that might lead to the removal of phenolic hydroxyl groups from the flavonoid parent nucleus and the opening of aromatic rings. Poor site selectivity and low catalytic efficiency are also the drawbacks of the reaction. Therefore, the reaction is commonly used for the synthesis of symmetrical bioflavonoids [58]. Suzuki coupling is currently the mainstream reaction for the synthesis of different kinds of biflavonoids. However, flavonoid boronic acid/boronic ester substrates are prone to boron removal and hydrolysis. Poor adaptability exists in the biflavonoids with high steric hindrance. Sonogashira synthesis is applicable to asymmetric flavonoids with specific connection modes, but it requires harsh reaction conditions and offers low yielding outcome. Stille synthesis is limited by the high toxicity residue of aryltin reagents. Chemical oxidative coupling typically relies on strong oxidants that promote side reactions and result in low yields. Enzymatic synthesis of biflavonoids only efficiently act on the flavonoid substrates with specific structures and substituents [6]. Additionally, the enzymes are difficult to isolate, purify or express heterologously, resulting in exorbitant acquisition costs.

To address these limitations, several aspects might focus on regulating the regioselectivity of coupling reactions and novel catalysts. Strategies such photocatalysis and electrocatalysis need to be explored to replace traditional coupling for improving reaction efficiency [59,60]. Regulating side products, flow chemistry can effectively address the prominent issues of limited mass transfer and excessive by-product formation in the traditional synthesis of biflavonoids, thereby significantly improving the reaction selectivity and conversion efficiency. In terms of biosynthesis, the substrate scope can be expanded by mining more dimeric enzymes and modifying the already known biflavonoid dimeric enzymes through directed evolution. Meanwhile, the metabolic pathways of microbial chassis such as *Escherichia coli* and *Saccharomyces cerevisiae* can be engineered; heterologous expression of key flavonoid synthases and dimeric enzymes enables the microbial biosynthesis of biflavonoids. Moreover, integrating enzymatic catalysis with chemical synthesis allows for the efficient chemical synthesis of flavonoid monomers, followed by the large-scale production of biflavonoids via catalysis with dimeric enzymes [61]. Computational tools and artificial intelligence are increasingly being employed to accelerate the discovery of metal catalysts and biocatalysts with tailored ligand architectures.

### 4.2. Biological Activities

Despite advances in the biological activity of biflavonoids, the translation from basic research to clinical application is scarce. The obstacles for biflavonoids include poor pharmacokinetic properties, the pleiotropy of their mechanisms of action and unclear targets, as well as insufficient safety and toxicity evaluation. Most biflavonoids have high lipophilicity and poor solubility, leading to inadequate gastrointestinal absorption, rapid hepatic metabolism, and failure to reach effective plasma concentrations. BBB penetration is also limited to obstacle the development in neurodegenerative diseases.

Intervention of medicinal chemistry is necessary to optimize the natural-origin biflavonoids and discover highly active derivatives with improved solubility and metabolic stability. Prodrug design and targeted delivery strategies can also be developed for rational drug design against specific diseases. Technologies such as liposomes and cyclodextrin inclusion can enhance the stability of biflavonoids, facilitating gastrointestinal absorption and targeted delivery. Unclear binding targets of these biflavonoids represent a major bottleneck hindering clinical translation. Thus, designing molecular probes based on biflavonoids, integrating chemical biology, bioinformatics analysis, and structural biology to uncover their therapeutic targets in specific diseases, will provide a solid foundation for subsequent precision drug development. In summary, systematic pharmacodynamic, pharmacokinetic, and toxicological studies are required to promote the drug development of biflavonoids.

## 5. Conclusions

Biflavonoids are a unique class of natural polyphenolic dimers, stemming their value from diverse bioactivities and potential pharmaceutical applications. We systematically summarize the synthetic strategies and clarify the extensive biological activities of the biflavonoids, raising the challenges on synthesis, target identification, and drug development prospects. Future research will focus on four key areas: combining green synthesis with enzyme engineering, optimizing pharmacokinetics via targeted delivery, identifying core targets with molecular probes, and developing high-activity derivatives for neurodegeneration diseases, drug-resistant tumors, and refractory infections. Biflavonoids are a core natural medicine focus, with synthetic breakthroughs and in-depth structure–activity relationship analysis driving their clinical translation. These questions will no doubt attract further interest in the coming years. It is also expected that novel biflavonoid-based drugs will be discovered using modern drug discovery strategies and new chemical sources.

## Figures and Tables

**Table 1:** Anti-tumor activity of representative biflavonoids.

Name (Source)	Activity	Mechanism/Pathway	Type [Ref]
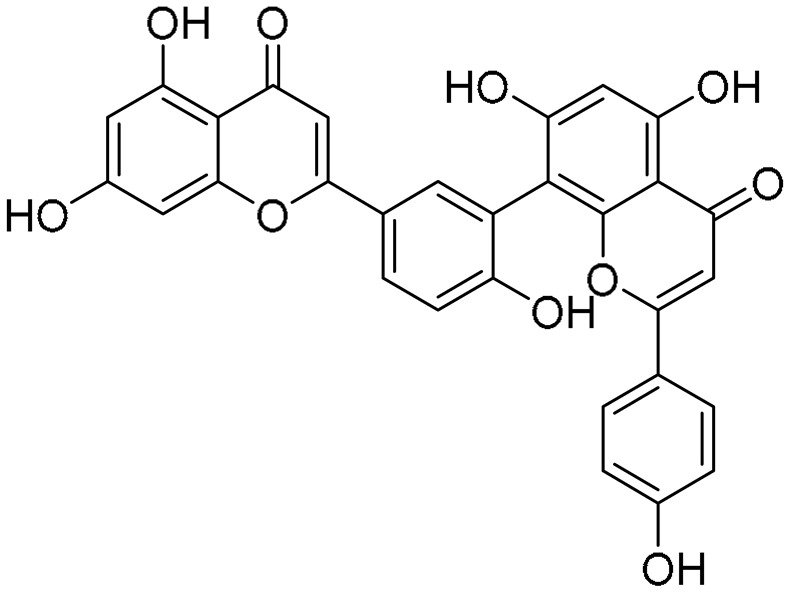 Amentoflavone (*Selaginella*)	AKR1B10 inhibitor (IC_50_ = 1.54 μM)	Blocking doxorubicin metabolism reverses resistance	Lung cancer [32]
	Treatment concentration: 50–100 μM	1. ROS/AMPK/mTOR pathway 2. Downregulating SLC7A11 GPX4	Endometrial carcinoma [33]
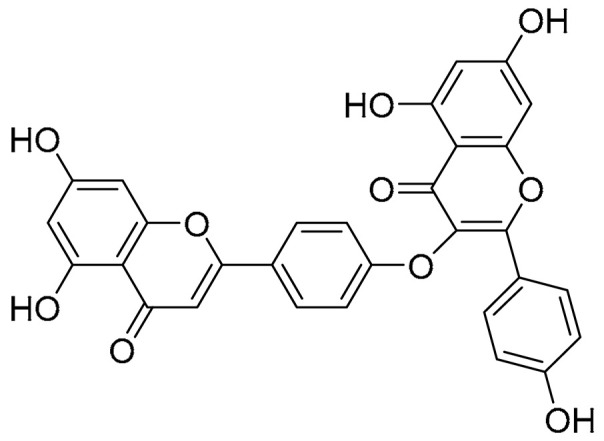 Delicaflavone (*Selaginella*)	Colorectal Cancer Cells, MTT Assay HT29 (IC_50_ = 18.8 μM)	1. In Vitro: PI3K/AKT/mTOR pathway, apoptosis 2. Ras/MEK/Erk signaling pathway 3. In Vivo: upregulating Caspase-3/9 and downregulating CD34/Ki67	Colorectal cancer [34]
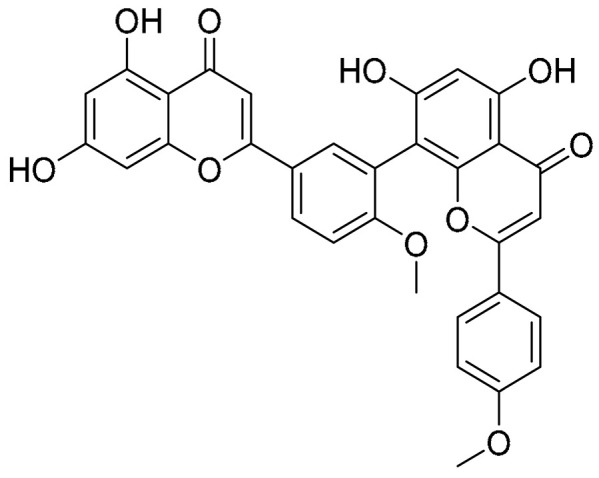 Isoginkgetin (*Ginkgo biloba*)	pre-mRNA splicing (IC_50_ = 30 μM)	Inhibiting pre-mRNA splicing	Anti-tumor activity [35]
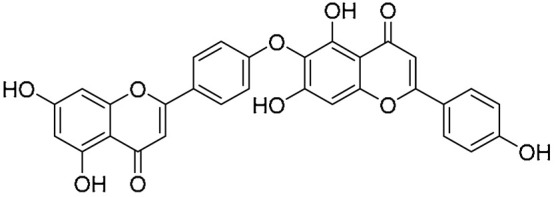 Hinokiflavone (*Platycladus orientalis*)	50 mg/kg (in vivo)	1. SIX4/Akt/Stat3 pathway 2. PI3K/AKT/mTOR and 3. Downregulating MMP2/9	Esophageal squamous cell carcinoma [36]
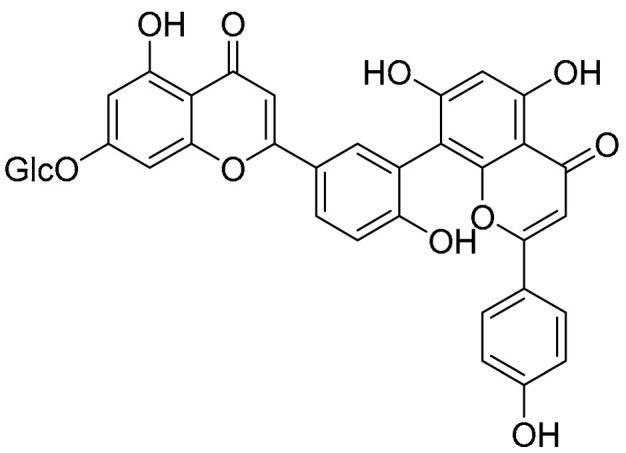 Amentoflavone-7-O-β-D-glucoside	Human prostate cancer PC-3 cells (IC_50_ = 57.7μM) Amentoflavone(IC_50_ = 102.2 μM)	Downregulating PDE6A, PDE10A	Human prostate cancer [7]

**Table 2:** Anti-Alzheimer’s disease activity of representative biflavonoids.

Name (Source)	Activity	Mechanism/Pathway	Ref
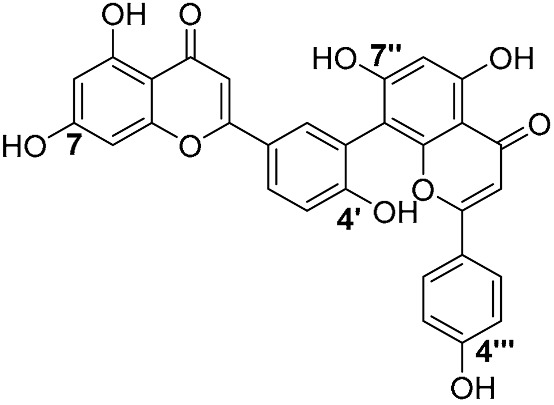 Amentoflavone (*Selaginella*)	Depolymerize Aβ_42_ fibrils (EC_50_ = 0.70 ± 0.16 μM) Inhibit Aβ_40_ aggegation (IC_50_ = 4.8 ± 0.1 μM)	1. Binding to the Aβ_42_ N-terminal pocket (4–14) via π-π stacking 2. Hydroxyl groups form hydrogen bonds with the backbone of Aβ peptides 3. Disrupting the β-sheet structure that maintains fibril stability	[37,38,39]
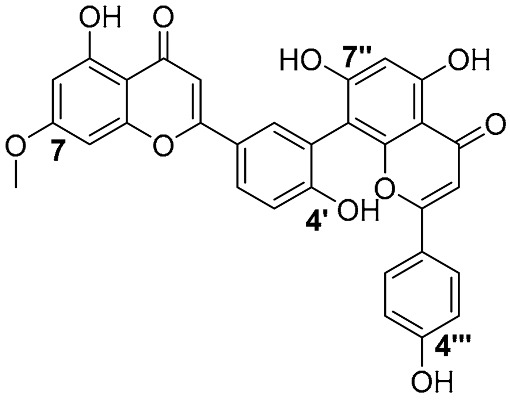 Sequiaflavone (*Selaginella*)	Depolymerize Aβ_42_ fibrils (EC_50_ = 1.89 ± 0.74 μM) Inhibit Aβ_40_ aggregation (IC_50_ = 4.9 ± 0.1 μM)		
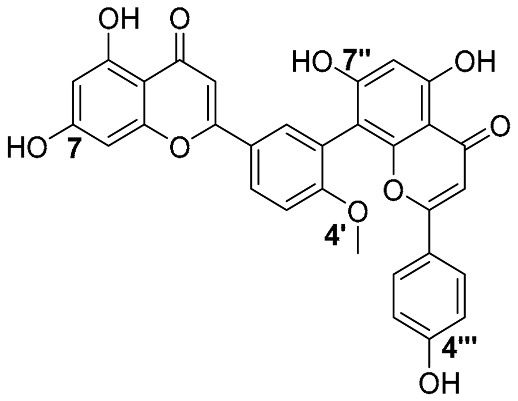 Bilobetin (*Selaginella*)	Depolymerize Aβ_42_ fibrils (EC_50_ = 2.75 ± 1.72 μM) Inhibit Aβ_40_ aggegation (IC_50_ = 4.7 ± 0.7 μM)		
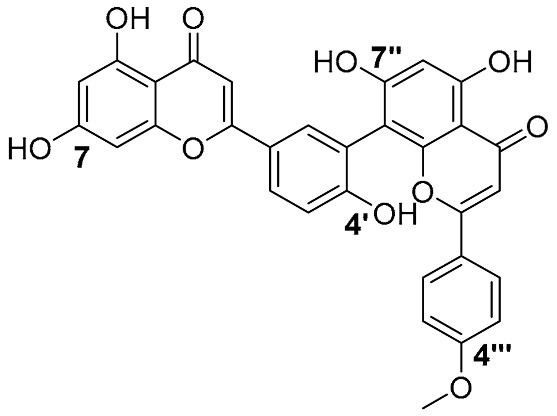 podocarpusflavone A (*Podocarpus neriifolius*)	Inhibit Aβ_40_ aggegation (IC_50_ = 4.9 ± 0.2 μM)		
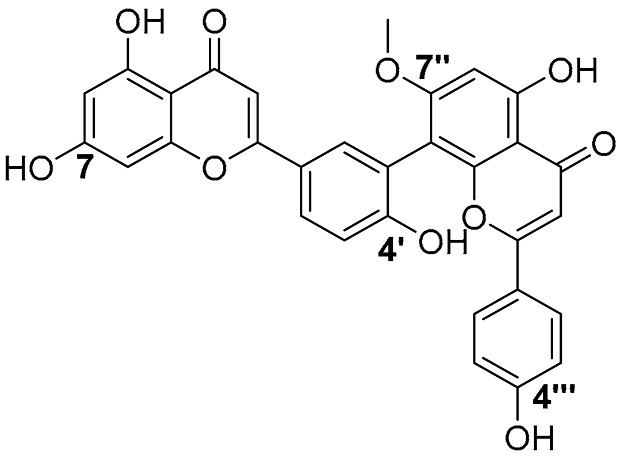 Sotetsuflavone (*Cycas revoluta*)	Inhibit Aβ_40_ aggregation (IC_50_ > 50 μM)		
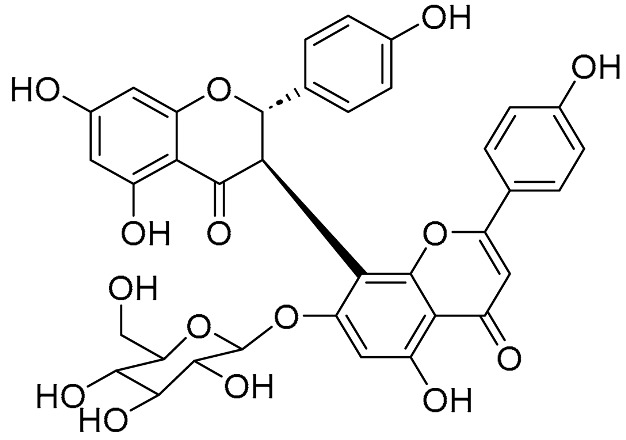 (2S,3S)-volkensiflavone-7-O-β-glucopyranoside (*Allanblackia floribunda*)	Inhibit MAO-A (IC_50_ = 35.85 ± 0.03 μM) Inhibit BACE-1 (IC_50_ = 2.48 ± 0.11 μM) Inhibit GSK-3β (IC_50_ = 9.39 ± 0.06 μM)	1. Neurotransmitter metabolic imbalance pathway (mediated by MAO-A) 2. β-amyloid (Aβ) deposition pathway (mediated by BACE-1) 3. Tau hyperphosphorylation pathway (mediated by GSK-3β)	[40]
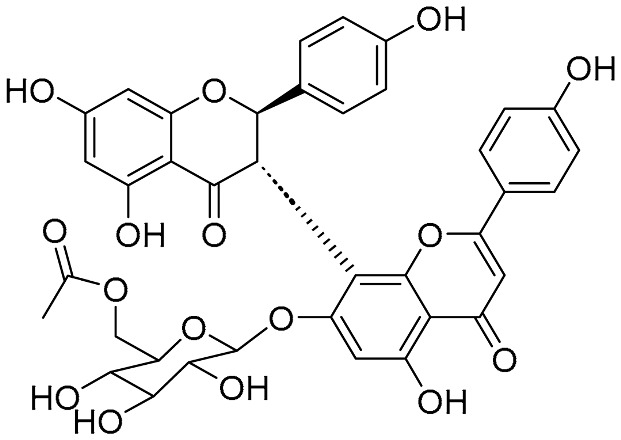 (2R,3S)-volkensiflavone-7-O-β-D-acetylglucopyranoside (*Allanblackia floribunda*)	Inhibit MAO-A (IC_50_ = 25.54 ± 0.05 μM) Inhibit BACE-1 (IC_50_ = 2.50 ± 0.17 μM) Inhibit GSK-3β (IC_50_ = 7.17 ± 0.09 μM)		

**Table 3:** Anti-inflammatory activity of representative biflavonoids.

Name (Source)	Activity	Mechanism/Pathway	Refs
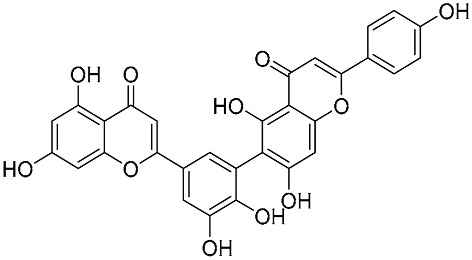 Robustaflavone (*Selaginella*)	Inhibit superoxide anion (IC_50_ = 1.01 μM) Inhibit elastase (IC_50_ = 0.45 μM)	1. Suppress superoxide anion generation 2. Inhibit elastase release	[41]
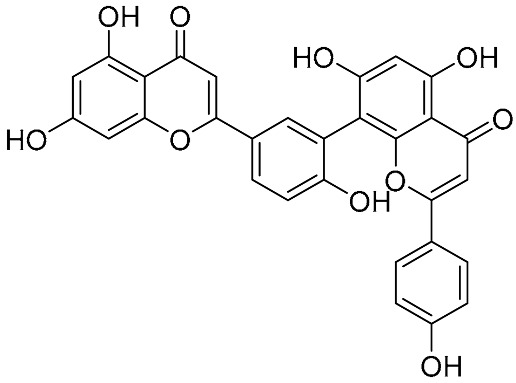 Amentoflavone (*Selaginella*)	Inhibit superoxide anion (IC_50_ = 1.01 μM) Inhibit elastase (IC_50_ = 0.75 μM)	1. Inhibit superoxide 2. Inhibit elastase	[41]
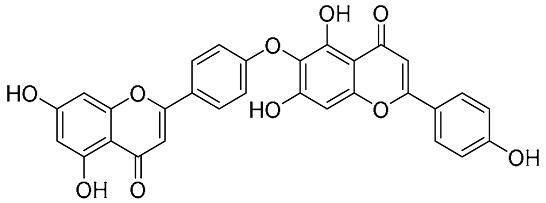 Hinokiflavone (*Platycladus orientalis*)	Range from 1 to 10 μM	1. ERK 1/2/NF-κB signaling pathway 2. Downregulate the iNOS and COX-2 3. regulate the SIX4/Akt/Stat3 pathway	[42,43]
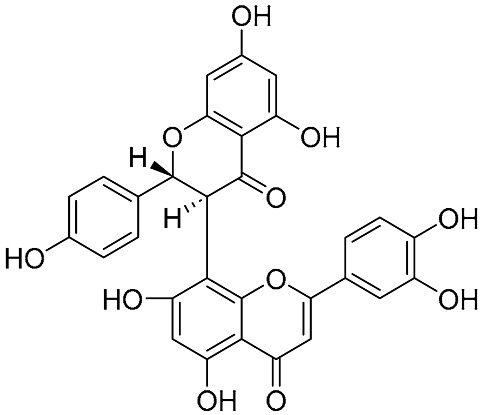 Morelloflavone (*Garcinia dulcis*)	II and III sPLA_2_ inhibitor (IC_50_ = 0.9 μM)	sPLA_2_ inhibition and ROS scavenging	[44]
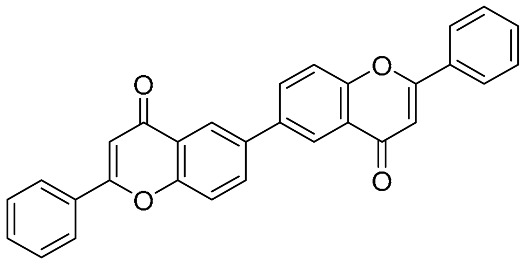 6,6″- linked biflavonoid (synthetic biflavonoids)	COX-2 inhibitor (IC_50_ = 3.7 μM)	Directly inhibit COX-2 and synergistically inhibit sPLA_2_-IIA	[45]
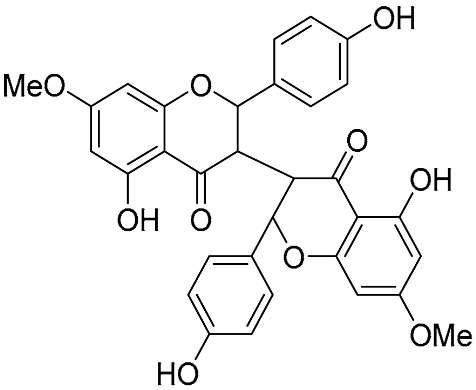 7,7″-di-O-methylchamaejasmin (*Khaya grandifoliola*)	Concentrations of 10, 20 μM and 40 μM	1. Block the formation of new ASC specks 2. Downregulate the gene expression of NFκB, NLRP3, and Caspase-1 3. Inhibit NO production and the secretion of IL-1β and IL-18	[46]
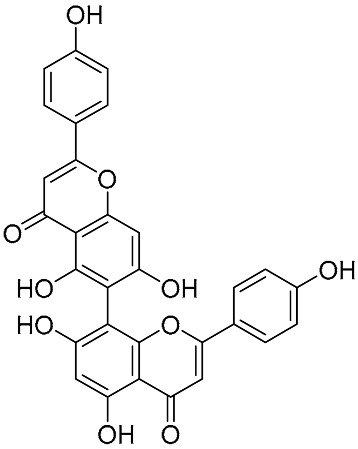 Agathisflavone (*Agathis palmerstonii*)	Concentrations of 0.1 μM and 1 μM	1. NF-κB signaling pathway 2. Downregulate TNF-α, IL-1β, CCL2, CCL5, NOS2 3. Upregulate IL-10 4. Reduce NO 5. Block M1 polarization of microglia 6. Inhibit microglial proliferation (reduce Iba-1^+^/BrdU^+^ cells	[47]

**Table 4:** Antimicrobial activity of biflavonoids.

Name (Source)	Activity	Mechanism/Pathway	Type [Refs]
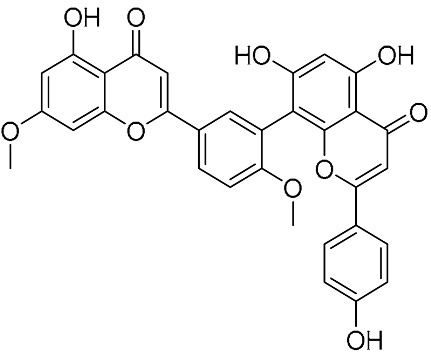 Ginkgetin (*Ginkgo biloba*)	Minimum biofilm inhibitory concentration: 6.25 µM (inhibition rate > 20%)	1. Reduce the production of AI-2 2. Downregulate the transcription of key genes (csgA, csgD, flhC, flhD, fliC, luxS, lsrB, lsrK, lsrR) 3. Inhibit EPS production	Antibacterial activity [48]
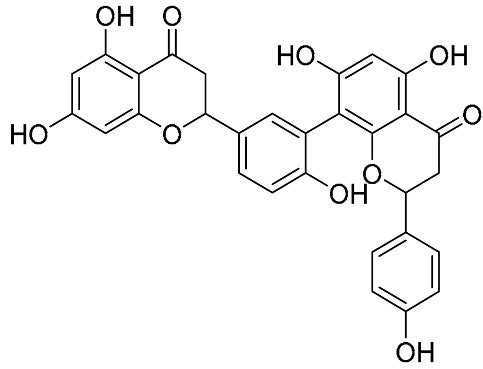 Tetrahydroamentoflavone (*Semecarpus prainii*)	1. MIC = 0.063 mg/mL 2. MBC = 0.063–0.125 mg/mL	Inhibit Gram-positive bacteria such as *Bacillus subtilis* and *staphylococcus carnosus*	Antibacterial activity [49]
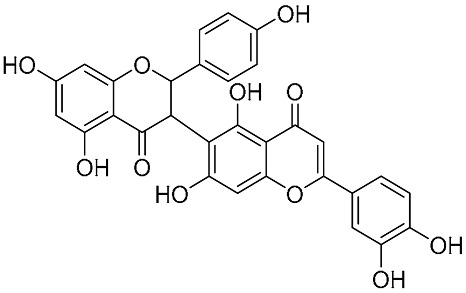 Macrophylloflavone (*Garcinia macrophylla*)	1. The inhibition zone for *Escherichia coli* ranges from 16.65 ± 0.43 mm to 20.29 ± 0.28 mm 2. For staphylococcus aureus, it ranges from 15.54 ± 0.39 mm to 23.16 ± 0.32 mm		Antibacterial activity [50]
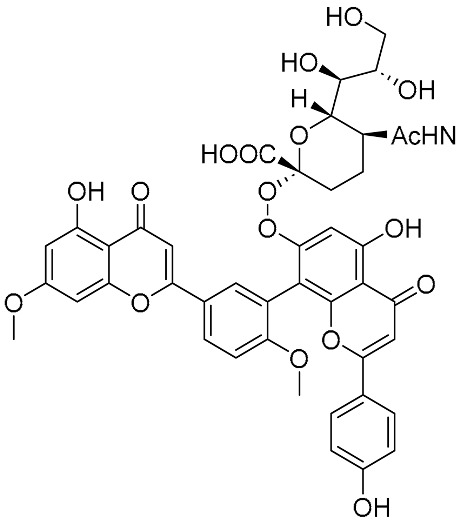 Ginkgetin derivatives (*Ginkgo biloba*)	1. H1N1 inhibitor (IC_50_ = 5.50 μg/mL) 2. H3N2 inhibitor (IC_50_ = 0.82 μg/mL)	1. Target influenza virus sialidase to block viral release and spread 2. Conjugation with sialic acid enables dual-site synergistic inhibition and reduces cytotoxicity	Antiviral activity [51]
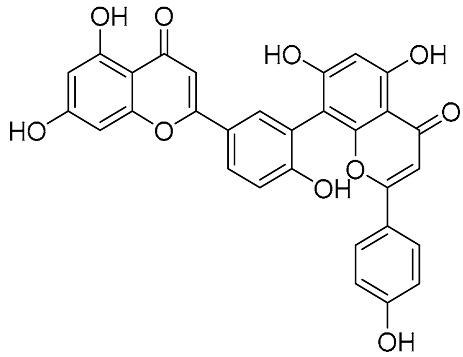 Amentoflavone (*Selaginella*)	1. RSV inhibitor (IC_50_ = 5.50 μg/mL) 2. 120 mg/kg/d	Inhibit RSV replication	Antiviral activity [52]
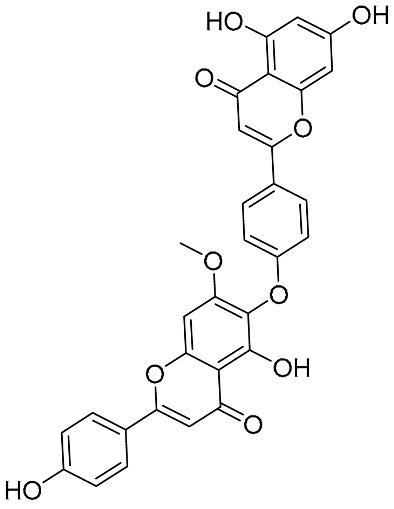 Isocryptomerin (*Selaginella tamariscina*)	MIC = 18.11 µM against *Candida albicans*, *Geotrichumcandidum*, *Saccharomyces cerevisiae*	Disruption of fungal cell membranes	Antifungal activity [53]

## Data Availability

No new data were created or analyzed in this study. Data sharing is not applicable to this article.

## Abbreviations

**AcOH** Acetic acid, **4CL** 4-Coumarate CoA Ligase, **AI-2** Autoinducer-2, **AKT** Protein Kinase B, **AMPK** Adenosine Monophosphate-Activated Protein Kinase, **BACE-1** β-Secretase 1, **BBB** Blood–Brain Barrier, **BCEC** Brain Capillary Endothelial Cells, **BrdU** 5-Bromo-2′-Deoxyuridine, **Caspase** Cysteine Aspartate-Specific Protease, **CCL** Chemokine (C-C Motif) Ligand, **CD34** Cluster of Differentiation 34, **CD68** Cluster of Differentiation 68, **CHI** Chalcone Isomerase, **CHS** Chalcone Synthase, **CI** Combination Index, **COX-2** Cyclooxygenase-2, **CRC** Colorectal Cancer, **CYP** Cytochrome P450, **DAT** Deamino tyrosine, **DCE** 1,2-Dichloroethane, **DIEA** N,N-Diisopropylethylamine, **DMSO** Dimethyl sulfoxide, **DMF** N,N-Dimethylformamide, **EPS** Extracellular Polymeric Substances, **ERK** Extracellular Regulated Protein Kinases, **ESCC** Esophageal Squamous Cell Carcinoma, **EtOAc** Ethyl acetate, **FNS I** Flavone Synthase I, **GPX4** Glutathione Peroxidase 4, **GSK-3β** Glycogen Synthase Kinase-3β, **HNE** Human Neutrophil Elastase, **Iba-1** Ionized Calcium Binding Adapter Molecule 1, **ID_50_** Inhibitory Dose 50, **IFN** Interferon, **IFNAR** Interferon Receptor, **iNOS** Inducible Nitric Oxide Synthase, **Ir(ppy)** Tris(2-phenylpyridine)iridium(III), **ISG** Interferon-Stimulated Gene, **Ki67** Ki-67 Antigen, **LPS** Lipopolysaccharide, **MAO-A** Monoamine Oxidase A, **MAVS** Mitochondrial Antiviral Signaling Protein, **MBC** Minimum Bactericidal Concentration, **MEK** Mitogen-Activated Protein Kinase, **MIC** Minimum Inhibitory Concentration, **MMP2/9** Matrix Metalloproteinase 2/9, **mTOR** Mammalian Target of Rapamycin, **NF-κB** Nuclear Factor-κB, **NLRP3** NOD-Like Receptor Pyrin Domain-Containing 3, **OMT** O-Methyltransferase, **PAL** Phenylalanine Ammonia-Lyase, **Papp** Apparent Permeability Coefficient, **PCNA** Proliferating Cell Nuclear Antigen, **PI3K** Phosphatidylinositol 3-Kinase, **QS** Quorum Sensing, **RIG-I** Retinoic Acid-Inducible Gene I, **ROS** Reactive Oxygen Species, **RSV** Respiratory Syncytial Virus, **SAR** Structure–Activity Relationship, **SLC7A11** Solute Carrier Family 7 Member 11, **sPLA_2_** Secretory Phospholipase A_2_, **TBAF** Tetra-n-butylammonium fluoride, **TBMSCl** tert-Butyldimethylsilyl chloride, **THF** Tetrahydrofuran, **TNF-α** Tumor Necrosis Factor-α, **TPA** 12-O-Tetradecanoylphorbol-13-Acetate
